# The influence of mycorrhizal hyphal connections and neighbouring plants on *Plantago lanceolata* physiology and nutrient uptake

**DOI:** 10.1007/s00572-025-01221-8

**Published:** 2025-08-02

**Authors:** Henry W. G. Birt, Lewis P. Allen, Sam Madge, Clare H. Robinson, Richard D. Bardgett, David Johnson

**Affiliations:** 1 Department of Earth and Environmental Sciences, The University of Manchester, Michael Smith Building, Manchester, M13 9PL UK; 2 Lancaster Environment Centre, Lancaster University, Bailrigg, Lancaster, LA1 4YQ UK

**Keywords:** Photosynthesis, Net ecosystem exchange, Common mycorrhizal network, Grassland, Carbon, Phosphorus, Magnesium, Zinc, Copper, Sulphur

## Abstract

Most plants extend their zone of interaction with surrounding soils and plants via mycorrhizal hyphae, which in some cases can form common mycorrhizal networks with hyphal continuity to other neighbouring plants. These interactions can impact plant health and ecosystem function, yet the role of these radial plants in mycorrhizal interactions and subsequent plant performance remains underexplored. Here we investigated the influence of hyphal exploration and interaction with neighbouring mycorrhizal plants, plants that are weakly mycorrhizal, and a lack of neighbouring plants on the performance of *Plantago lanceolata*, a mycotrophic perennial herb common to many European grasslands, using mesh cores and the manipulation of neighbouring plant communities. Allowing growth of hyphae beyond the mesh core increased carbon capture above-ground and release below-ground as root exudates and resulted in the greater accumulation of elements relevant to plant health in *P. lanceolata*. However, contrary to expectations, the presence of mycorrhizal, or weakly mycorrhizal neighbours as well as an absence of neighbours did not significantly alter the benefits of hyphal networks to *P. lanceolata*. Our findings demonstrate that enabling the development of a fungal network beyond the immediate host rhizosphere significantly influences plant leaf elemental stoichiometry, enhances plant carbon capture, and increases the amount of carbon they release via their roots as exudates. Our experimental design also provides a simple set of controls to prevent attributing positive mycorrhizal effects to neighbouring plant connections.

**Supplementary Information:**

The online version contains supplementary material available at 10.1007/s00572-025-01221-8.

## Introduction

Arbuscular mycorrhizal (AM) fungi form symbiotic relationships with the vast majority of plant species where they play a crucial role in various plant processes including nutrient uptake (Read and Perez-Moreno 2003; Wipf et al. 2019) and stress tolerance (Bunn et al. 2009; Hohmann and Messmer 2017; Bitterlich et al. 2018). More broadly, these AM fungal-plant interactions can mediate nutrient cycling, thereby affecting ecosystem function (Yu et al. 2023). For example, mycorrhiza can affect both photosynthetic processes and the subsequent release of carbon into the soil as root exudates (Gavito et al. 2019; Muneer et al. 2020; Xu et al. 2023). This dual effect is likely to result in increased carbon capture by the plant and a corresponding increase in carbon release below-ground due to the carbon demands of the mycorrhizal symbiosis. However, how mycorrhizal fungi modify the interconnectedness of carbon uptake above-ground and its release below-ground as root exudates remains unclear. Elucidating how mycorrhiza may alter carbon cycling is important because the release of plant-derived carbon below-ground is a primary driver of microbial metabolism in soil (Fan et al. 2022). Soil microbes other than mycorrhiza are also responsible for essential functions, including plant growth promotion, nutrient cycling, and pathogen protection (Wen et al. 2021; Fan et al. 2022). Furthermore, soil carbon contributes to several important abiotic functions, such as maintaining soil structure and moisture, as well as preventing soil erosion (Zhang et al. 2024). Understanding the role of mycorrhiza in carbon cycling is also essential for developing accurate climate models, as below-ground carbon storage mediated by these fungi represents a significant, yet not fully quantified, carbon sink (Hawkins et al. 2023).

Carbon cycling is a crucial aspect of mycorrhizal function, yet the influence of these fungi on the accumulation of other nutrients is equally significant. While the role of AM fungi in phosphorus and nitrogen uptake is well-documented (Smith and Read 2008), research suggests that they may also play a role in the cycling of other essential elements, such as sulphur and calcium (Allen and Shachar-Hill 2009; Fu et al. 2023). Furthermore, mycorrhiza can mediate the uptake and accumulation of heavy metals, which can have profound impacts on plant health and ecosystem functions (Rosas-Moreno et al. 2023). The differential uptake of these nutrients and elements, mediated by mycorrhizal associations, can alter plant tissue chemistry, affecting herbivory, decomposition rates, and ultimately, biogeochemical cycling (Hartley and Gange 2009). It is therefore likely that mycorrhiza will influence the accumulation of a broad range of minerals, yet quantitative assessment of the accumulation of a range of minerals simultaneously in plants subjected to mycorrhizal treatments is lacking in the current literature (Sardans et al. 2023). Therefore, to gain a holistic view of their ecological significance, a more comprehensive understanding and reporting of how mycorrhizal fungal networks influence the acquisition of a broader range of elements—beyond the common focus on phosphorus and nitrogen—is crucial.

An emergent property of AM fungi-plant relationships is the development of hyphal networks. The structure and function of these networks is important in the transport of nutrients from areas that plant roots cannot access (Wipf et al. 2019). They can also provide a physical connection between individual plants of the same or different species, forming common mycorrhizal networks (CMNs) (Alaux et al. 2021). These networks can mediate competition between adult plants (Lin et al. 2015; Tedersoo et al. 2020; Bahadur et al. 2023), and have varying effects (positive, negative, or neutral) on establishing seedlings (Van Der Heijden and Horton 2009). Fungal hyphae can also interact with neighbouring plants without forming a direct physical connection. Therefore, common mycorrhizal networks that exhibit direct hyphal connections (CMN-HC) between roots represent a specific subset within the broader category of fungal networks (Rillig et al. 2024). Thus, it follows that the functioning of a host plant may be affected when its extra-radical hyphae can interact with neighbouring plants regardless of whether hyphal continuity occurs.

Various studies have demonstrated that involvement in a CMN can alter plant uptake and allocation of nutrients. For instance, when connected via a CMN, flax (*Linum usitatissimum*) obtained substantial nutrients for minimal carbon, while sorghum (*Sorghum bicolor*) acquired few nutrients despite a large carbon investment (Walder et al. 2012). When these species were combined in a CMN flax gained biomass without the loss of biomass from sorghum thus demonstrating that a CMN can maximise resource efficiency within a plant community. Interspecies dynamics has shown to influence CMN nutrient dynamics in other species as well (Graves et al. 1997; Bougoure et al. 2014). While CMN-HCs have the potential to facilitate resource transfer between plants, the presence of other plants may also alter CMN dynamics through the creation of nutrient gradients, production of inhibitory and stimulatory chemicals (Barto et al. 2011), and competition for fungal resources (Wang et al. 2022). These effects could be substantial, and may also be caused by non-mycorrhizal plants, which can still compete for resources and alter the soil environment (Lambers and Teste 2013). Yet, it remains unclear how non-mycorrhizal plants relative to mycorrhizal plants influence neighbouring plants via interaction with CMNs. Nevertheless, it is likely that these effects could be substantial and mediated by whether neighbouring plants form mycorrhizas or not. Furthermore, when investigating the role of CMNs, it is important to consider the total volume of soil that mycorrhizal fungi can explore within a system (Karst et al. 2023). When AM fungi are limited to resources within a smaller soil volume, they have far less nutrients available to forage than in a larger soil volume. Therefore, differences in plant performance may be attributable to a ‘soil foraging volume effect’. This potential effect has been highlighted for field-based experiments on ectomycorrhizal fungi, but also applies to other mycorrhizal types including AM fungi (Karst et al. 2023).

To address these knowledge gaps, we explored how the extension of extra-radical mycorrhizal hyphae, and their interaction with different neighbouring plants, influence plant physiology and nutrient uptake. To achieve this, we conducted a greenhouse experiment using *Plantago lanceolata* as a model plant. It is a mycotrophic perennial herb common to European grasslands that has been extensively used as a model for mycorrhizal interactions. *P. lanceolata* was grown inside mesh containers that allowed fungal hyphae, but not roots, to grow outward. The cores were placed inside larger pots (mesocosms) that contained one of three treatments: (1) *P. lanceolata* plants (mycorrhizal); (2) *Rumex acetosa* plants (a weakly mycorrhizal species); or (3) fallow (no plants). This design allowed us to manipulate the connections via extra-radical mycorrhizal mycelium to the outside of the core by rotating half of the cores once per week. We then assessed how these treatments (outer compartment species and connection by extra-radical mycelium) influenced a range of plant attributes including biomass, chlorophyll fluorescence, gross ecosystem exchange of carbon and primary production, and leaf elemental composition. We hypothesised that plants connected to an outer soil compartment via extra-radical mycorrhizal mycelium would exhibit increased carbon exchange compared to unconnected plants. In addition, we expected connection to an outer soil compartment via extra-radical mycorrhizal mycelium to alter the pattern of accumulation of a broad range of elements in the connected plant compared to unconnected plants. Together with increased carbon assimilation, we expected this alleviation of nutrient limitation to result in increased plant biomass. Finally, we hypothesised that focal plants neighbouring other mycorrhizal plants (potentially forming CMN-HC) would demonstrate enhanced nutrient uptake compared to focal plants neighbouring plants that rarely make mycorrhizal associations or surrounded by bare soil because of the increased resource use efficiency afforded by CMNs.

## Methods

### Mesocosms and greenhouse conditions

Soil for the greenhouse experiment was collected from the top 20 cm from land adjacent to Formby Beach, Liverpool, UK (Latitude: 53.56113; Longitude: −3.08637). The area sampled is a semi-natural dune grassland dominated by *Ammophila arenaria* but which also included *Plantago lanceolata*. The sandy soil facilitated the sampling of root exudates with minimal damage to the root system when removing the plant from growing in soil to hydroponics. Yet, the soil contained natural mycorrhizal inoculum, which was confirmed through a preliminary pilot trial. The soil was passed through a sieve (8 mm) and homogenised.

Sieved soil was placed into 26 L pots (43 cm x 34 cm x 18 cm). An empty cylindrical core, measuring 12 cm in height with 5 cm diameter with 4 cm x 8 cm windows cut in the side, was covered in nylon mesh with a 40 μm aperture and placed into the centre of each pot. The 40 μm mesh allows penetration by AM fungal hyphae but prevents penetration by roots and rotation of the mesh disrupts fungal hyphae (Johnson et al. 2001).

*Plantago lanceolata* seeds (Emorsgate seeds, King’s Lynn, UK) were placed 2.5 cm apart in between two sheets of heavy weight seed germination paper then rolled up and placed vertically in deionised water and incubated in the dark at room temperature for 5 days. Following germination, seedlings were placed into the outer compartment of the pots approximately 5 cm apart for an initial soil conditioning stage to provide the sole source of mycorrhizal inoculum for plants growing in the central mesh core; this resulted in 24 plants outside of the core. Plants were grown for six weeks and then removed.

**Fig. 1:**
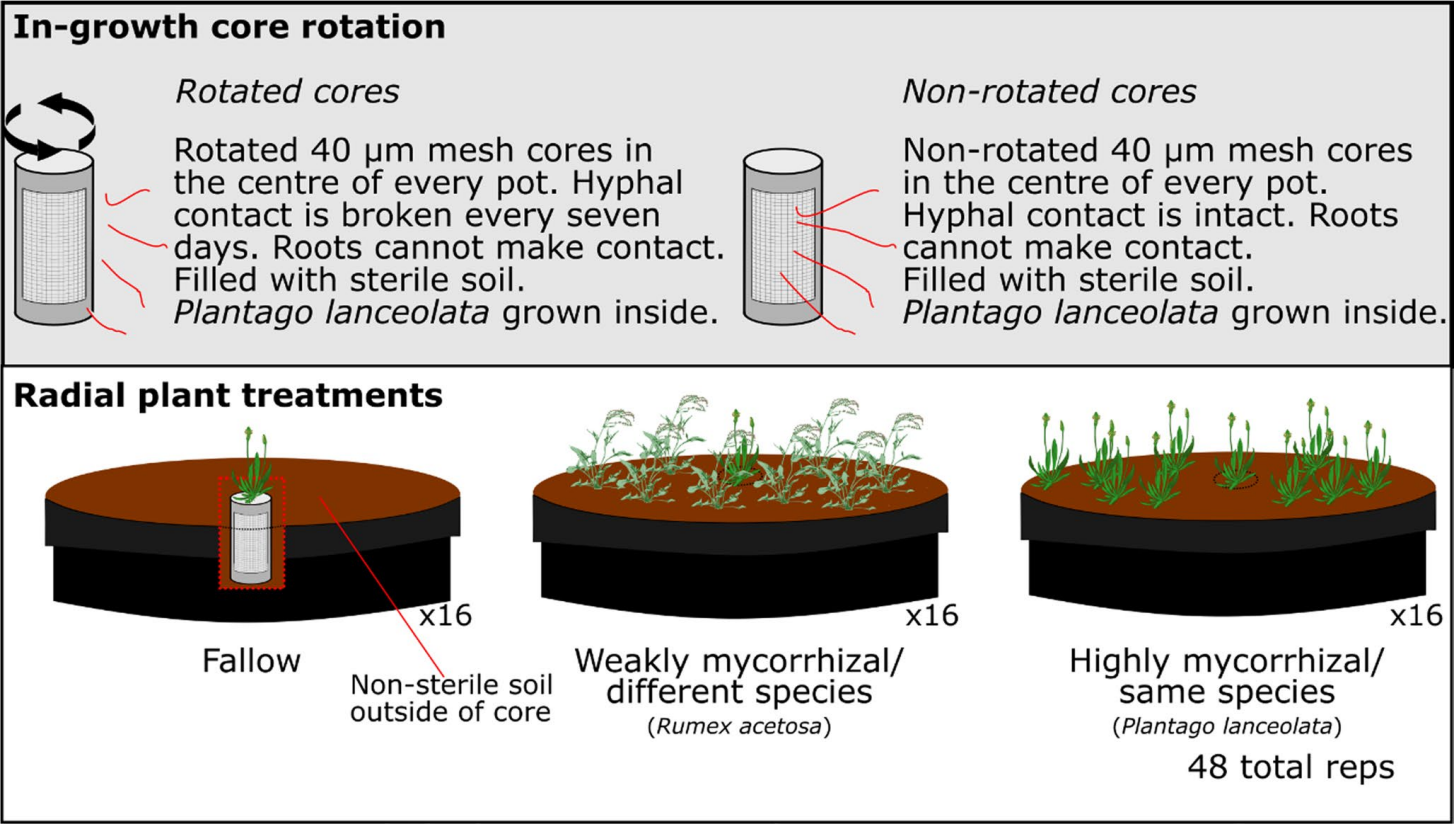
A schematic of the experimental design in this study

Three planting treatments were then applied to the outer (‘radial’) compartment of the conditioned soils (*Plantago lanceolata* [mycorrhizal], *Rumex acetosa* [although generally regarded as non-mycorrhizal, we refer to it as ‘weakly mycorrhizal’], and fallow, i.e., no plants) (Fig. 1). Radial plants were placed into pots at the same rate as the conditioning stage. The central core was then filled with 230 g of autoclaved soil and three *Plantago lanceolata* seedlings were planted into the core (referred to hereafter as the core plant). Autoclaved soil was added to the centre to ensure mycorrhizal colonisation of the central plants occurred by growth of extra-radical mycelium from outside of the core, likely from a CMN, but potentially from independent spores. Throughout the experiment cores were rotated approximately 45° once per week in half of all pots to sever mycorrhizal hyphal connections (Johnson et al. 2001). Pots were arranged in a randomised block design to account for any differences attributable to environmental variation in the greenhouse.

Plants grew for two weeks and were then thinned to the single strongest plant. Growing conditions were maintained at 14–25 °C and sixteen hours of light, with supplementary lighting to replicate summer conditions in the UK for all growing periods. To prevent soil moisture loss and to protect the seedlings from soil pests, a 2 cm depth of perlite was spread across the soil surface. Soil moisture was maintained every three days at 60% water holding capacity (WHC). WHC was measured using a soil moisture probe (Theta probe ML3 attached to a HH2 Moisture Meter, Delta-T Devices, Cambridge, UK). Each pot was an independent replicate, and eight pots were allocated per treatment for a total of 48 replicates across all treatments (Fig. 1).

### Net ecosystem carbon exchange

After seven weeks of growth, measurements of gross CO_2_ exchange were made from the core plant over 135 s intervals with a PP systems EGM5 portable IRGA coupled to a cylindrical chamber, 7 cm diameter and 19 cm height (Ward et al. 2007, 2013). One chamber was transparent to permit light infiltration and fitted with lights to ensure even light between all replicates, and the other chamber was opaque. These contrasting chamber configurations enabled the measurement of gross CO_2_ flux through subtracting ecosystem respiration (dark conditions) from net CO_2_ flux (light conditions).

### Sampling procedure

Following CO_2_ exchange measurements, the core plants were checked for signs of stress using a FluorPen FP100. The FluorPen quantifies chlorophyll A by measuring the intensity of red fluorescence (685 nm) emitted after blue light excitation (470 nm), with lower fluorescence indicating reduced chlorophyll concentration, plant stress, and potentially decreased photosynthetic rates (Padhi et al. 2021). Three FluorPen measurements were taken on the second emerged leaf of the plant that had previously been darkened by being wrapped in aluminium foil for at least one hour. Plants were then carefully removed from the core and roots were washed under tap water. Biomass was separated into above and belowground parts. The roots were dabbed dry with paper towel and weighed wet; a proportion were then weighed and separated for an assessment of AM fungal root colonisation and stored in 70% ethanol at 4 °C. The remaining biomass was then dried at 65 °C for 24 h and weighed. The amount of dried biomass removed for AM fungal root colonisation was calculated through the loss of water weight from the remaining roots. The roots of ten *Rumex* plants were randomly sampled and checked for AM fungal colonisation.

### Root exudate sampling and analysis

Six replicates from every treatment were subjected to root exudate sampling. Root exudates were collected according to the method of Williams et al. (2021). Briefly, plants were removed from pots and loosely adhered soil was removed. Roots were separated from the remaining soil by submerging the plants 3–5 times in a beaker of deionised water until the water appeared clear. Plants were then allowed to recover for three days in 150 mL of soil hydroponics solution aerated using an aeration stone and air pump. Plants were maintained under the same greenhouse conditions as the previous stage of the experiment. The soil hydroponic solution was created by combining 200 g of soil from the same source that had been used for the pot experiment with 1 L of water. This mixture was placed on an orbital shaker for 1 h at 90 rpm and then centrifuged at 3,200 RCF for 5 min and the supernatant kept. After three days of recovery, plants were washed again in deionised water and placed into 150 mL of aerated fresh Milli-Q water on ice under the same greenhouse conditions. After two hours of collection, plants were removed, and the remaining solution was passed through a 0.22 μm filter. After root exudates had been collected, the plants were sampled in the same manner as other plants. A preliminary trial was conducted to check the impact of root exudate collection on histological observations of root structures and no significant difference was found. Root exudates were analysed for total C on a Shimadzu TOC-L CPN E200 TOC analyser (Kyoto, Japan).

### Quantification of AM fungal root colonisation and hyphal length

The prevalence of AM fungal structures within root segments was evaluated using a magnified gridline intersections method (McGonigle et al. 1990; Vierheilig et al. 1998). Following ethanol removal, root segments were immersed in 10 mL of 10% KOH at room temperature for 1 h 10 min to facilitate clearing. Subsequently, the cleared samples were rinsed with deionised water, subjected to acidification by submersion in 1% HCl for 30 s, and then transferred to test tubes containing 10 mL of 1% Parker’s Quink Blue ink solution in 1% HCl at room temperature for 1 h. Excess stain was removed by thorough washing with water. Finally, the stained root segments were transferred to 15 mL falcon tubes containing a de-staining solution composed of glycerol, water, and lactic acid in a 1:1:1 ratio. Following incubation in the de-staining solution for at least 24 h, 5 root segments of 4 cm in length were mounted onto microscope slides using lactoglycerol (1:1:1 lactic acid, glycerol, and water) as a mounting medium. For each slide, 50 root-graticule intersections were examined to determine the proportion containing visible fungal hyphae, arbuscules, and vesicles.

A membrane filter technique was used to measure hyphal length (Hanssen et al. 1974). Briefly, a 2 g sample of soil from the central core was mixed with 500 mL dH_2_O for 2 min at high speed with a magnetic stirrer. A 200 mL sub-sample was then decanted from the top, span again, and then counter-stirred to allow large particles to sink. A 30 mL sub-sample was then filtered through 25 mm diameter, 1.2 μm pore size, white polycarbonate filters (Cytiva, MA, USA) placed on top of a Buchner funnel held under vacuum. The filter was then incubated with a 5% Parker’s Quink Blue ink and 5% acetic acid solution for 5 min and then rinsed three times with 5 mL dH_2_O. Filters were mounted on slides with a 50% glycerol solution. Using a LEICA DM2500 microscope at 100x magnification, gridded sections were counted on a filter until the variation in the mean number of hyphal intersections varied by < 1% three times in a row. Hyphal length (H) was calculated as H = (IπA)/(2 L), where I = average intersections per grid, A = grid area, and L = total grid line length. Finally, total fungal hyphae length (F) in mm mg^−1^ of soil was determined using F = H × (A/B)*(1/S), where A = filter area, B = grid area, and S = soil amount filtered.

### Leaf elemental composition

To assess leaf elemental composition, 50 mg of dried leaf biomass was weighed into glass acid-washed test tubes. For each digestion a set of 38 tubes, 2 tubes of blanks and 2 tubes of hay powder as a certified reference material (Commission of the European Communities, Community Bureau of Reference, reference material 129), to assess recovery rates, were also prepared. To each tube, 0.42 mL of concentrated nitric acid (70%) was added and left overnight to release nitrogen dioxide. Subsequently, 0.42 mL of hydrogen peroxide (30%) was added, and the tubes were placed in a heat block at 100 °C for 1 h, then 120 °C for a further hour, and finally 140 °C for 2 h. Samples were then diluted to 2% of HNO_3_ using MiliQ water and syringe filtered using a 0.22 μm filter. These solutions were run on an ICP-OES, Agilent 5800, using a 1.2 kW Argon RF plasma at 12 L min^−1^ and auxiliary gas running at 1 L min^−1^. Sample concentrations were analysed and reported as the average of three by five-second integrations. Sample uptake into the ICP-OES was done at 0.7 L min^−1^ using a Mira-Mist nebuliser.

### Statistical analyses

Univariate variables were assessed using mixed-effects linear models with experimental block as a fixed effect; this analysis was implemented through the R package *lme4* (Bates et al. 2015). Post-hoc analysis was conducted using the R package *emmeans* through analysis of estimated marginal means with Benjamin-Hochberg corrections (Lenth 2023). The *vegan* R package was used to assess multivariate significant differences between leaf elemental composition by testing Euclidean distance between samples using PERMANOVA (Oksanen et al. 2022).

## Results

### Chlorophyll fluorescence and biomass

Plants across all treatments exhibited chlorophyll fluorescence values indicative of healthy plants (~ 0.8, Fig S1, Zhori et al. 2015).

**Fig. 2:**
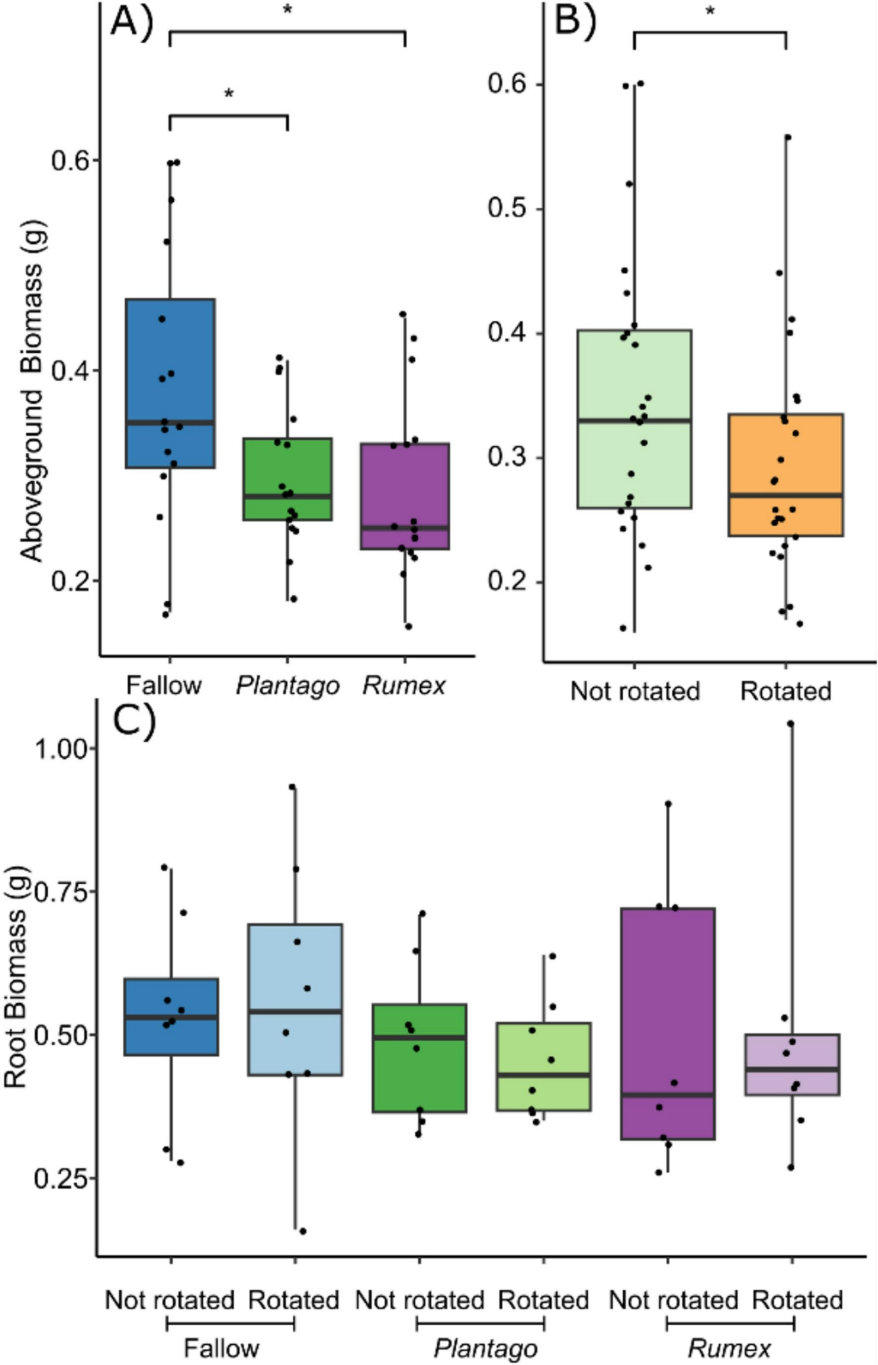
The impact of the experimental treatments on plant biomass. (**A**) The radial plant community and (**B**) the rotation of an in-growth core on dry leaf biomass as well as (**C**) their interaction on the dry root biomass of *Plantago lanceolata*. Boxplots show the range, median, and interquartile range of each treatment. Dots overlaid show individual data points. Significance between treatments is shown: *** < 0.001, ** < 0.01, and * < 0.05

The rotation of the core influenced above-ground biomass, but did not influence root biomass of the core plants. Rotated plants had 15.4% less above-ground biomass than those with static cores. Plants without any radial plants (fallow) also had more above-ground biomass than plants surrounded by either *R. acetosa* or *P. lanceolata* (on average, 25.1% and 22.0%, respectively; Fig. 2). However, radial plants had no influence on root biomass. There was no observed interaction between core rotation and radial plant community (Table 1).

**Table 1:**
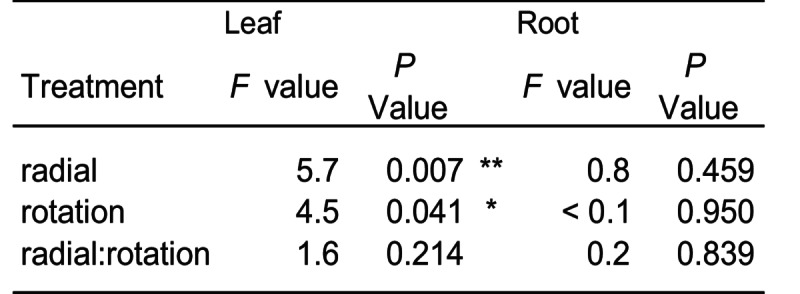
The effect of radial plant community (radial) and core rotation (rotation) on the biomass of *Plantago lanceolata*

### Leaf elemental composition

**Fig. 3:**
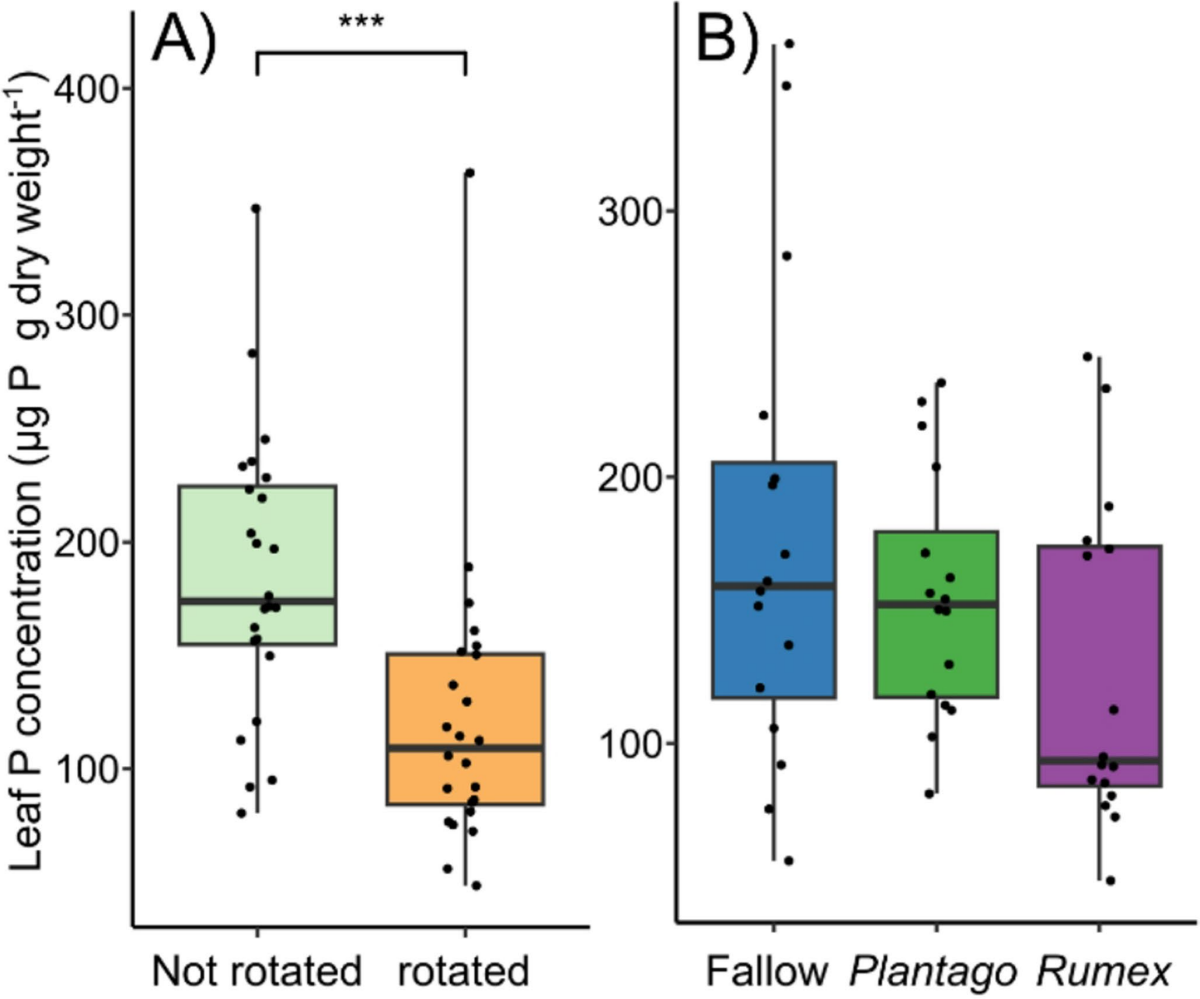
The impact of the experimental treatments on phosphorus accumulation. The effect of (**A**) the rotation of an in-growth core and (**B**) the radial plant community on phosphorus leaf content in *Plantago lanceolata*. Boxplots show the range, median, and interquartile range of each treatment. Dots overlaid show individual data points. Significance between main effect of rotation treatment is shown: *** < 0.001, ** < 0.01, and * < 0.05

The elemental composition of leaves was influenced by the rotation of the in-growth core; however, radial plants had no influence on plant leaf elemental composition and no interaction was observed between the treatments (Table 2, Fig S2). Leaf concentrations of phosphorus, a key element foraged by mycorrhiza, were 51% higher on average in plants grown in static cores (Fig. 3). Radial plants did not influence leaf phosphorus concentration (Fig. 3). In addition, magnesium, sulphur, copper, and zinc were all elevated in plants grown in static cores (Table 3.

**Table 2:**
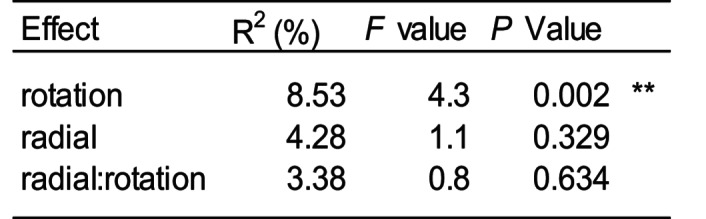
PERMANOVA results showing the effect of radial plant community (radial), and core rotation (rotation) on the multivariate elemental composition of the leaves of *Plantago lanceolata*

**Table 3:**
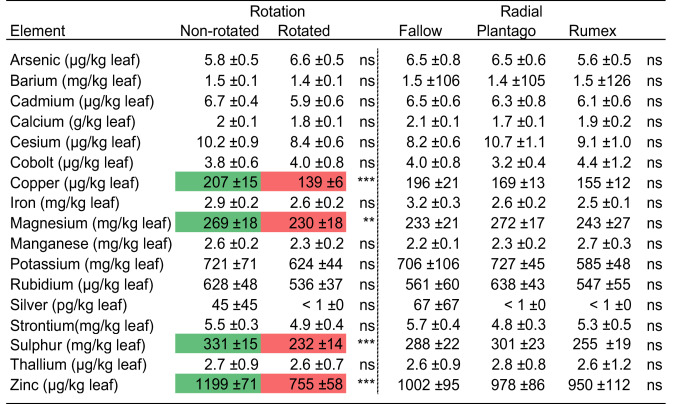
The effect of radial plant community (radial) and core rotation (rotation) on the mean elemental composition of the leaves of*Plantago lanceolata*. Significantly highest mean values are coloured green, and the lowest values red. ± indicates the standard error of the mean

### Impacts on above- and below-ground carbon flux

**Fig. 4:**
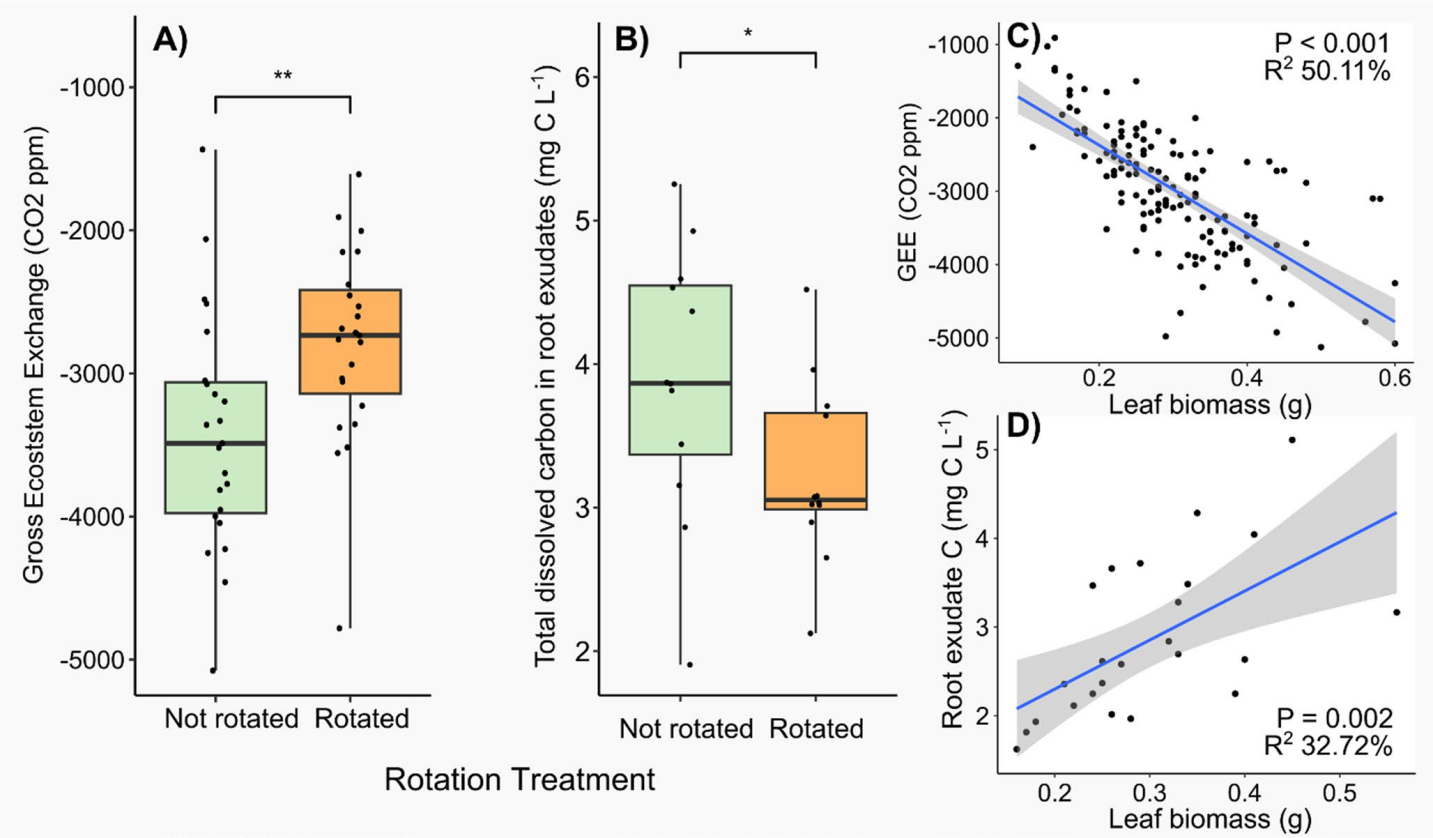
The impact of the experimental treatments on carbon exchange. The effect of the rotation of an in-growth core on (**A**) gross ecosystem exchange and (**B**) total carbon in root exudates of *Plantago lanceolata.* (**C**) The relationship between gross ecosystem exchange and (**D**) total dissolved carbon in root exudates with leaf biomass. Boxplots show the range, median, and interquartile range of each treatment. Dots overlaid show individual data points. Significance between treatments is shown: *** < 0.001, ** < 0.01, and * < 0.05

Plants grown in static cores had a significantly lower (18.2% less on average) gross ecosystem exchange (GEE) compared to those in rotated cores (Table 4; Fig. 4), indicating greater fixation of carbon by plants in static cores. Radial plant community did not influence GEE. In addition, rotation of the mesh core surrounding the plant significantly reduced the total amount of carbon exudated by plant roots by 24.2% on average (Table 4; Fig. 4). No significant impact of radial plants on the total amount of carbon exudated by plant roots was detected. There was a significant correlation between GEE and the total amount of carbon in root exudates with biomass (Fig. 4). When GEE and the total amount of carbon in root exudates were scaled by above-ground biomass (CO_2_ g^−1^ dry biomass and mg C L^−1^ g^−1^ dry biomass, respectively), they were no longer significant (Table S1, Fig. S3). There was no significant impact on soil respiration (Fig S4).

**Table 4:**
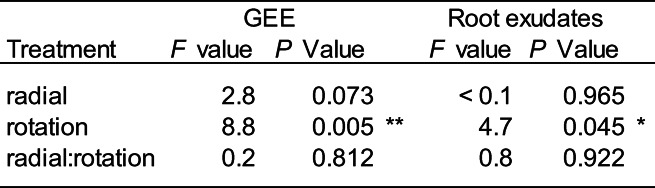
The effect of radial plant community (radial) and core rotation (rotation) on the total amount of carbon exudated and gross ecosystem exchange (GEE) of *Plantago lanceolata*

### AM fungal colonisation

**Fig. 5:**
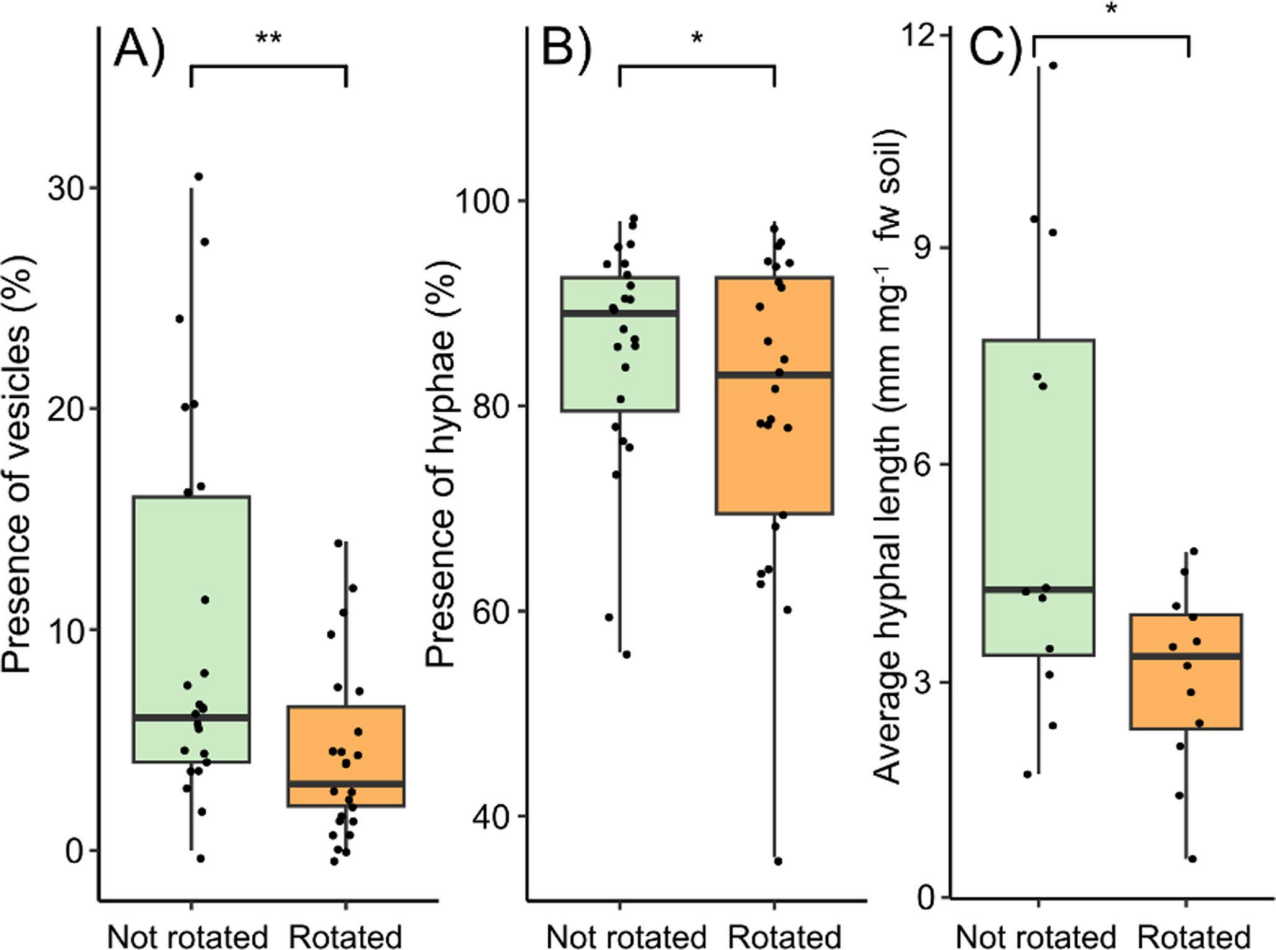
The impact of the experimental treatment on fungal colonisation. The effect of the rotation of the in-growth core on the presence of (**A**) vesicles and (**B**) hyphae in the roots of *Plantago lanceolata* as well as (**C**) the impact of core rotation on the average hyphal length found in the soil within the core. Boxplots show the range, median, and interquartile range of each treatment. Dots overlaid show individual data points. Significance between treatments is shown: *** < 0.001, ** < 0.01, and * < 0.05

No evidence of mycorrhizal colonisation was observed in any radial *Rumex* roots sampled, confirming many previous observations that *Rumex* spp. are weakly mycorrhizal. Although all *P. lanceolata* roots from the mesh cores were colonised by mycorrhiza, fungal hyphae and vesicles were significantly more abundant in the roots that were in static as opposed to rotated cores. Radial plants, regardless of species, did not influence the presence of hyphae or vesicles in the roots (Table 5; Fig. 5). No treatment influenced the presence of arbuscules (Table 5). Hyphal length in soil from inside the core was significantly greater in static compared to rotated cores (Table 5; Fig. 5). Radial plants did not influence hyphal length.

**Table 5:**
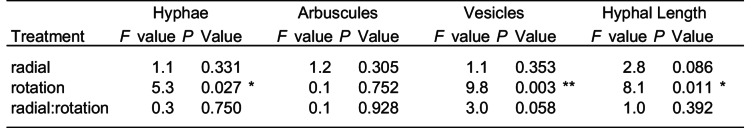
The effect of radial plant community (radial), and core rotation (rotation) on root associated AM fungal structures in Plantago lanceolata. Asterisks indicate significant differences

## Discussion

This study investigated how the extension of extra-radical mycorrhizal hyphae, and their interaction with different neighbouring plants, influence the physiology and nutrient uptake of *Plantago lanceolata*. Enabling growth of hyphae into the radial compartment increased plant carbon capture above-ground and release below-ground as root exudates, and resulted in the greater accumulation of elements key to plant health, especially phosphorus, copper, sulphur, and zinc; although 13 other elements remained unaffected by the treatments. However, contrary to expectations, the presence of mycorrhizal or weakly mycorrhizal neighbours did not significantly alter the benefits *Plantago lanceolata* derived from having greater exploratory extra-radical mycorrhizal hyphae. This finding highlights the importance of hyphal exploration of soil regardless of the presence of a simple radial plant community.

### Biomass and carbon dynamics

Plants that had hyphae that could penetrate the radial compartment had greater above-ground biomass than those that could not (Fig. 2). This was in line with our expectation that mycorrhiza would alleviate nutrient limitation resulting in greater plant biomass. Other studies have found that association with AM fungi can promote (Zhang et al. 2015), have no impact (Karasawa et al. 2012), or decrease biomass of *P. lanceolata* (Qu et al. 2021). These differing results may arise from differences among studies in soil conditions, especially nutrient availability, and AM fungal genotypes. Here, we prioritised sourcing AM fungi from an environment where *P. lanceolata* grew wild, as the AM fungal community would likely be adapted to promoting *P. lanceolata*. Although AM fungal association has been reported to promote below-ground biomass (Zhang et al. 2015), no significant impacts were detected in our study, although there was a promotion of above-ground biomass. This may have been due to the mesh cores restricting growth, although no evidence of roots being pot bound was apparent at sampling. The promotion of above-ground biomass may be due to the additional carbon sink from the connected mycorrhizal hyphae, that in turns stimulates leaf growth to supply additional carbon (Kaschuk et al. 2009). This theory is in-line with our hypothesis that mycorrhiza would stimulate carbon absorption and release below-ground as root exudates and was confirmed by our experimental results (Fig. 4). Other literature also supports that disconnection from mycorrhizal hyphae reduces plant photosynthetic rate (Gavito et al. 2019). These findings has implications for the role that AM fungi play in terrestrial carbon cycling (Hawkins et al. 2023).

Our findings confirmed our hypothesis that plant connection to a more extensive AM fungal network induces an increase in photosynthetic rate and this also caused an increased quantity of root exudate carbon. This finding therefore connects above-ground and below-ground effects of mycorrhizal symbiosis that have been reported separately (Gavito et al. 2019; Muneer et al. 2020; Xu et al. 2023). We observed a strong positive correlation between leaf biomass and both above-ground carbon capture and below-ground carbon release as root exudation (Fig. 4). This suggests that the effect is mediated by an increase in photosynthetic tissue as opposed to some metabolic shift. Nevertheless, the observed enhancements in photosynthesis and below-ground carbon allocation may be constrained in environments where mycorrhizal networks cannot provide sufficient resources to support increased biomass. This limitation of growth could occur in nutrient-poor soils, such as used here, or when other environmental factors, such as temperature, restrict plant growth.

### Nutrient uptake

Plants grown in static cores had, on average, a 51% higher concentration of phosphorus in their leaves, likely due to the connection to mycorrhizal hyphae outside of the core (Fig. 3). This supports the well-established role that mycorrhiza play in plants obtaining phosphorus (Smith and Read 2008; Jiang et al. 2021; Tibbett et al. 2022). In addition, magnesium, sulphur, copper, and zinc were all elevated (Table 3), which are key to plant health. For example, magnesium is the central atom in the chlorophyll molecule (Shaul 2002); sulphur is a key constituent in some amino acids (Hawkesford and De Kok 2006); copper serves as a cofactor for electron-transfer proteins (Burkhead et al. 2009); and zinc is a co-factor in thousands of proteins, including those involved in CO_2_ fixation (Broadley et al. 2007). Therefore, this study adds to evidence that mycorrhiza help plants to accumulate a range of elements that are essential to their survival (Allen and Shachar-Hill 2009; Lehmann and Rillig 2015; Zare-Maivan et al. 2017; Rosas-Moreno et al. 2021). The accumulation of these elements in plants due to mycorrhizal interactions could have important implications for plant-herbivory interactions (Balluffi-Fry et al. 2022), and the nutritional status of crops (Lehmann and Rillig 2015).

### Mycorrhizal colonisation

The presence of vesicles and hyphae in roots, as well as the average hyphal length inside the in-growth core was reduced in rotated cores (Fig. 5). This indicates that the rotation treatment disrupted connections between the focal plant and mycorrhiza outside of the core. Vesicles and hyphal density are often used as proxies of performance of mycorrhizal fungi but do not always correlate with performance benefits for the plant (Gange and Ayres 1999), although in our experiment plant performance was better in the static treatments. Arbuscule formation was not different between treatments, although these structures are transient (2–8 days) and the sampling represents a single time point sampling (Luginbuehl and Oldroyd 2017). Therefore, differences in arbuscules present in the root may have been more apparent at different stages of the symbiosis.

### Lack of radial plant effects

We found no significant effect of the presence or identity of radial plants on the biomass of the central *P. lanceolata* grown inside mesh cores This lack of response occurred regardless of whether the neighbour was a mycorrhizal plant of the same species (i.e. *P. lanceolata*) or a weakly mycorrhizal plant of a different species (i.e. *Rumex acetosa*). This outcome contradicts our hypothesis that a radial community of mycorrhizal plants would improve plant performance by enhancing resource use through a CMN. Our findings instead suggest that soil foraging effects are important, whereby plant biomass increases when its mycorrhizal network is given access to a greater volume of soil e.g., the soil outside the in-growth core (Karst et al. 2023). A limitation of our study, as with many manipulations of CMNs, is that we could not establish the extent that a continuous functional CMN-HC had formed, which typically requires use of isotopic tracing (Lekberg et al. 2024). Nevertheless, our dual controls (weakly mycorrhizal neighbours and fallow) prevented us attributing observed positive effects on *P. lanceolata* of non-rotated cores versus rotated cores to CMNs. The weakly mycorrhizal plants were used because they likely alter soil conditions similarly to mycorrhizal plants but cannot form a CMN-HC, whereas the fallow treatment prevents both CMNs, and the specific circumstance of CMN-HC. This methodological approach is important, as techniques like isotopic tracing can be restrictive due to cost, accessibility, and regulations, especially in field experiments, and are not necessarily equivocal in identifying the presence of CMN-HC. While our controls prevented misattributing positive effects to a CMN, further work would be required to definitively attribute the effects to a CMN-HC. The need for extensive controls is critical in this field of research. A recent review by Lehmann and Rillig (2024) found that only 8.3% of published studies met the stringent criteria for defining a functional CMN-HC, underscoring the necessity of the robust controls utilised in our experiment.

It is possible that the duration of plant growth in this study (seven weeks) was insufficient to establish a CMN-HC. Although an initial condition phase was included, the removal and replacement of plants may have disrupted an established CMN. Furthermore, other studies have suggested that CMN effects may be most evident when there are imbalances within the network, such as differences in carbon costs to a plant for mycorrhiza-obtained nutrients (Walder et al. 2012), or plant access to water (Egerton-Warburton et al. 2007). Our study had similar genotypes growing in a relatively homogeneous environment. As such, biological market theory would suggest that with no variation in supply or demand (even soil resources), and no partner choice driving competition (similar genotype), a single, uniform “exchange rate” (the price of nutrients for photosynthate) would emerge across the entire population, thus with little trading advantage to be gained (Bunn et al. 2024). Future studies may consider designs with likely imbalances in the CMN partners.

Our findings also counters the hypothesis that non-mycorrhizal plants may induce CMN-like effects (Wang et al. 2022) as well as our hypothesis that radial mycorrhizal plants would modulate mycorrhizal effects. This is evident as we did not observe an impact of radial *R. aceotosa* on plant performance in the static cores relative to those with no radial plants or those with *P. lanceolata* as radial plants. However, the inclusion of non-mycorrhizal plants in a greater range of environmental conditions and plant communities is required to fully establish their role in CMN function. Beyond nutrient transfer, other CMN effects, such as herbivory signalling, may have been present, but were not tested in this study (Babikova et al. 2013).

## Conclusion

This study demonstrates that enabling the development of a fungal network beyond the immediate host rhizosphere significantly enhances carbon capture by plants and increases the amount of carbon they release at their roots as exudates, and that this effect is strongly correlated with increases in leaf biomass. Furthermore, our results indicate that mycorrhiza facilitate plant accumulation of a range of elements in addition to phosphorus, especially micronutrients zinc, copper, and sulphur. Critically, this study also underscores the importance of including non-mycorrhizal and no-plant controls to accurately attribute plant performance to CMN effects, rather than mycorrhizal soil foraging effects.

## Electronic supplementary material

Below is the link to the electronic supplementary material.

Supplementary Material 1 (DOCX 357 KB)

## Data Availability

No datasets were generated or analysed during the current study.
