# Supplementary material for: The influence of mycorrhizal hyphal connections and neighbouring plants on Plantago lanceolata physiology and nutrient uptake
Source: Mycorrhiza. 2025 Aug 2;35(4):48. doi: 10.1007/s00572-025-01221-8 (PMC12317909; doi:10.1007/s00572-025-01221-8)
Supplement: Supplementary file 1 — Supplementary Material 1 (DOCX 357 KB) [file 572_2025_1221_MOESM1_ESM.docx]

**
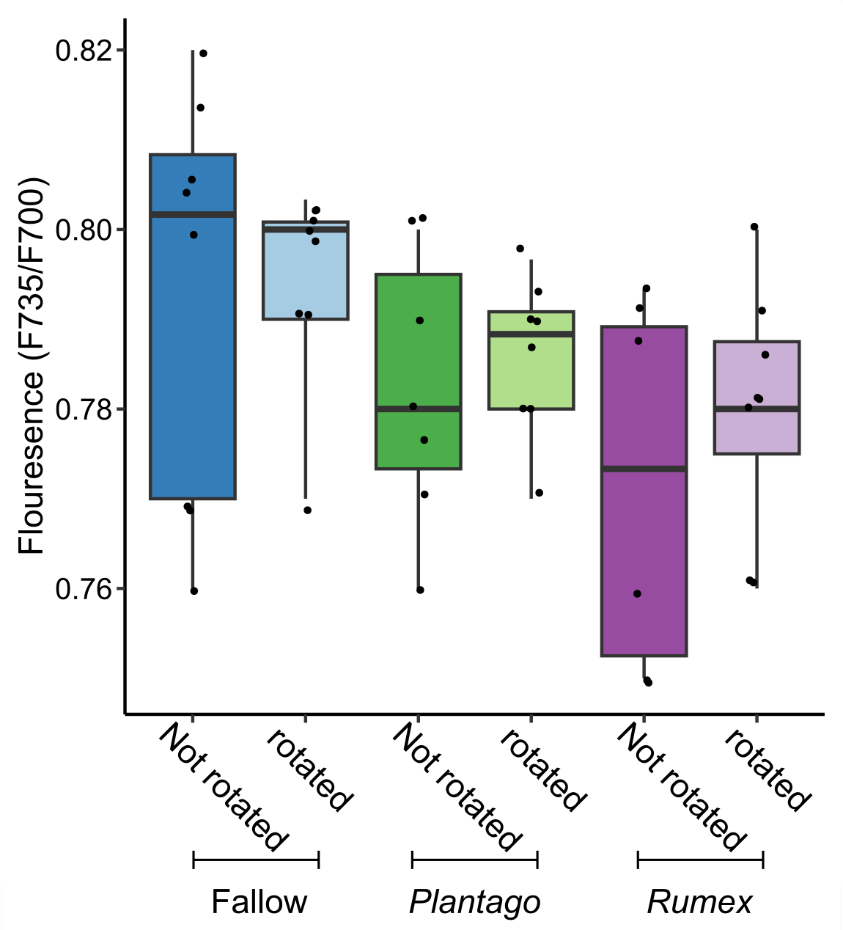
**

**Fig. S1** The effect of plant radial community and the rotation of an in-growth core on the chlorophyll fluorescence of *Plantago lanceolata*.


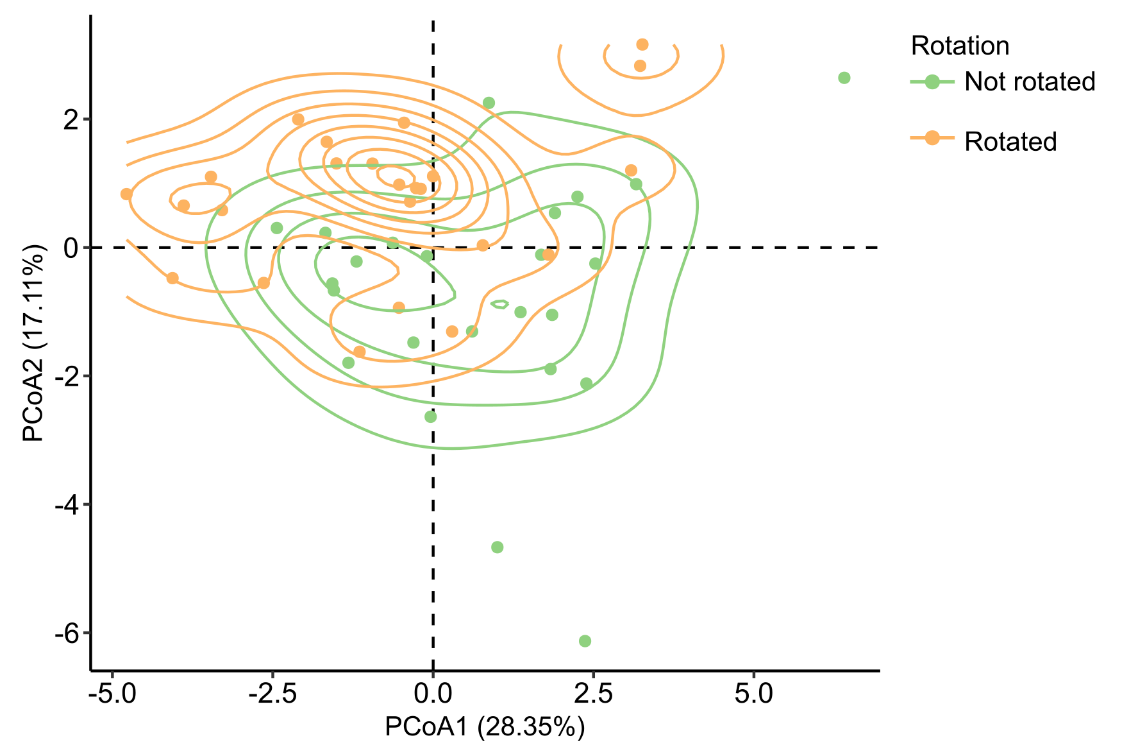


**Fig S2** A plot of principal coordinate analysis of Euclidean distance between elemental composition of the leaves of *Plantago lanceolata*. Points are coloured by the effect of the rotation of the in-growth core. Contour lines represent two-dimensional kernel density showing an estimation of probability density. Box plots on the x and y axis show the primary and secondary axis scores between treatments.

**Table S1** The effect of radial plant community (radial) and core rotation (rotation) on the total amount of carbon exudated and gross ecosystem exchange (GEE) per gram of dry biomass of *Plantago lanceolata*.

**
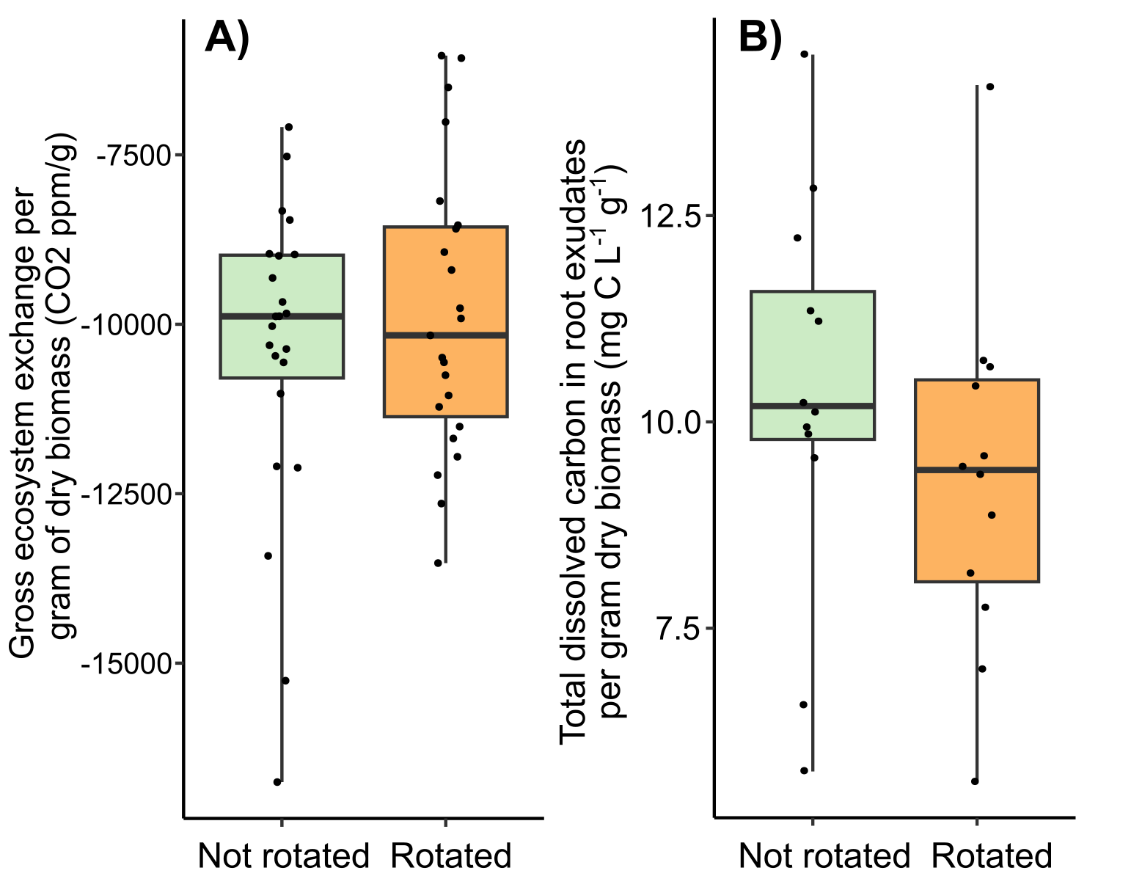
**

**Fig. S3** The effect of the rotation of an in-growth core on A) net ecosystem exchange and B) total carbon in root exudates per gram of dry biomass of *Plantago lanceolata*. Boxplots show the range and interquartile range of each treatment. Dots overlaid show individual data points. Significance between treatments is shown: *** < 0.001, ** < 0.01, and * < 0.05.

**
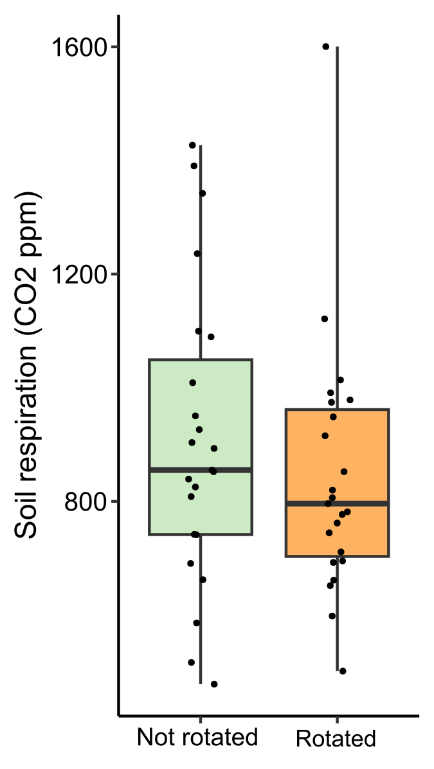
**

**Fig. S4** Impact of in-growth core rotation on soil respiration in *Plantago lanceolata* mesocosms. Boxplots show the range and interquartile range of each treatment. Dots overlaid show individual data points.
